# Gastric *Helicobacter suis* Infection Partially Protects against Neurotoxicity in A 6-OHDA Parkinson’s Disease Mouse Model

**DOI:** 10.3390/ijms222111328

**Published:** 2021-10-20

**Authors:** Helena Berlamont, Arnout Bruggeman, Eva Bauwens, Charysse Vandendriessche, Elien Clarebout, Junhua Xie, Sofie De Bruyckere, Griet Van Imschoot, Elien Van Wonterghem, Richard Ducatelle, Patrick Santens, Annemieke Smet, Freddy Haesebrouck, Roosmarijn E. Vandenbroucke

**Affiliations:** 1 Department of Pathobiology, Pharmacology and Zoological Medicine, Faculty of Veterinary Medicine, Ghent University, 9820 Merelbeke, Belgium; Helena.Berlamont@UGent.be (H.B.); Eva.Bauwens@UGent.be (E.B.); Sofie.DeBruyckere@UGent.be (S.D.B.); Richard.Ducatelle@UGent.be (R.D.); Freddy.Haesebrouck@UGent.be (F.H.); 2 VIB Center for Inflammation Research, 9052 Ghent, Belgium; Arnout.Bruggeman@irc.VIB-UGent.be (A.B.); Charysse.Vandendriessche@irc.VIB-UGent.be (C.V.); Elien.Clarebout@irc.VIB-UGent.be (E.C.); Junhua.Xie@irc.VIB-UGent.be (J.X.); Griet.VanImschoot@irc.VIB-UGent.be (G.V.I.); Elien.VanWonterghem@irc.VIB-UGent.be (E.V.W.); 3 Department of Biomedical Molecular Biology, Ghent University, 9052 Ghent, Belgium; 4 Department of Neurology, Ghent University Hospital, 9000 Ghent, Belgium; Patrick.Santens@ugent.be; 5 Laboratory of Experimental Medicine and Pediatrics, Faculty of Medicine and Health Sciences, University of Antwerp, 2610 Antwerp, Belgium; Annemieke.Smet@uantwerpen.be

**Keywords:** Parkinson’s disease, gut–brain axis, oxidative stress, *Helicobacter*, 6-OHDA

## Abstract

The exact etiology of Parkinson’s disease (PD) remains largely unknown, but more and more research suggests the involvement of the gut microbiota. Interestingly, idiopathic PD patients were shown to have at least a 10 times higher prevalence of *Helicobacter suis* (*H. suis*) DNA in gastric biopsies compared to control patients. *H. suis* is a zoonotic *Helicobacter* species that naturally colonizes the stomach of pigs and non-human primates but can be transmitted to humans. Here, we investigated the influence of a gastric *H. suis* infection on PD disease progression through a 6-hydroxydopamine (6-OHDA) mouse model. Therefore, mice with either a short- or long-term *H. suis* infection were stereotactically injected with 6-OHDA in the left striatum and sampled one week later. Remarkably, a reduced loss of dopaminergic neurons was seen in the *H. suis/*6-OHDA groups compared to the control/6-OHDA groups. Correspondingly, motor function of the *H. suis*-infected 6-OHDA mice was superior to that in the non-infected 6-OHDA mice. Interestingly, we also observed higher expression levels of antioxidant genes in brain tissue from *H. suis*-infected 6-OHDA mice, as a potential explanation for the reduced 6-OHDA-induced cell loss. Our data support an unexpected neuroprotective effect of gastric *H. suis* on PD pathology, mediated through changes in oxidative stress.

## 1. Introduction

Parkinson’s disease (PD) is the second most common neurodegenerative disease after Alzheimer’s disease, affecting more than 6 million people worldwide in 2016 and exceeding 12–17 million by 2040 [1,2]. Although the exact etiology remains largely unknown, several precipitating factors, such as aging, hereditary factors, dietary factors, environmental pollution, and bacterial and viral infections were found to contribute to increased risk of developing PD [3,4]. PD clinical symptoms can be divided into motor symptoms and non-motor symptoms, with some types of non-motor symptoms (e.g., sleep disorders, hyposmia, constipation, depression) often preceding the onset of motor symptoms [5]. These symptoms and their time course parallel the major neuropathological hallmarks in PD which are the selective loss of dopaminergic neurons in the substantia nigra pars compacta and the presence of alpha-synuclein containing Lewy bodies in different nuclei of the nervous system [6].

There is increasing evidence that PD might be initiated in the gastrointestinal tract [7,8,9,10,11,12,13,14]. A gastric *Helicobacter* (*H*.) *pylori* infection has already been associated to an increased risk of developing PD, and also with worse motor function in established PD [15,16,17,18,19,20,21]. In addition, *H. pylori* infection may affect the bioavailability of levodopa, which is used for PD treatment, resulting in worse motor control in *H. pylori*-infected PD patients [22]. *H. pylori* eradication has been suggested to result in improvement of PD-related motor symptoms [16,17,19,23,24,25,26], although a recent randomized control study could not confirm this hypothesis [27].

Interestingly, in one study, an exceptional high frequency of *H. suis* DNA was found in gastric biopsies from patients with idiopathic PD compared to a non-PD control group (27% vs 2%) [28]. In addition, eradication of the *H. suis* infection in a PD patient resulted in a remarkable improvement of the gastric as well as the neurological symptoms [29]. *H. suis* is a zoonotic spiral-shaped non-*H. pylori Helicobacter* (NHPH) species naturally colonizing the stomach of pigs and non-human primates [30]. Transmission from pigs to humans may occur through direct or indirect contact between *H. suis*-infected pigs and humans. Raw or undercooked pork may also constitute a source of *H. suis* infection in humans, as *H. suis* can be present and survive in minced pork [31]. The ability of *H. suis* to cause extra-gastric effects was confirmed in a recent mouse study from our lab [32]. In this study, a short-term *H. suis* infection of 1 month was shown to not only induce gastric inflammation, but also to cause a disturbance of the gastrointestinal barrier integrity, low-grade systemic inflammation, disruption of the blood–cerebrospinal fluid (CSF) barrier, and brain inflammation accompanied by cognitive decline [32]. Clearly, the potential role of a gastric *H. suis* infection in neurodegenerative disorders, and PD in particular, requires further investigation.

The 6-hydroxydopamine (6-OHDA) model is often used as an experimental model for PD as it leads to its main pathological and clinical hallmarks [33]. The model’s relevance is also highlighted by the observation of 6-OHDA in the blood and urine of PD patients [34]. 6-OHDA is a structural analogue of catecholamines (i.e., dopamine and noradrenaline) and causes toxic effects to catecholaminergic neurons through the generation of reactive oxygen species (ROS) leading to the induction of oxidative stress and subsequent mitochondrial dysfunction and cell death [35,36]. After unilateral injection of 6-OHDA in the striatum, degradation of dopaminergic neurons occurs in a retrograde fashion in the nigrostriatal pathway [37,38]. 

To further investigate the potential role of *H. suis* in PD, short-term and long-term *H. suis*-infected mice were subjected to a unilateral injection with 6-OHDA in the left striatum. One week later, the effects of *H. suis* infection on PD-related hallmarks were evaluated.

## 2. Results

### 2.1. Successful Gastric H. suis Colonization Is Associated with Gastric Inflammation

**Figure 1:**
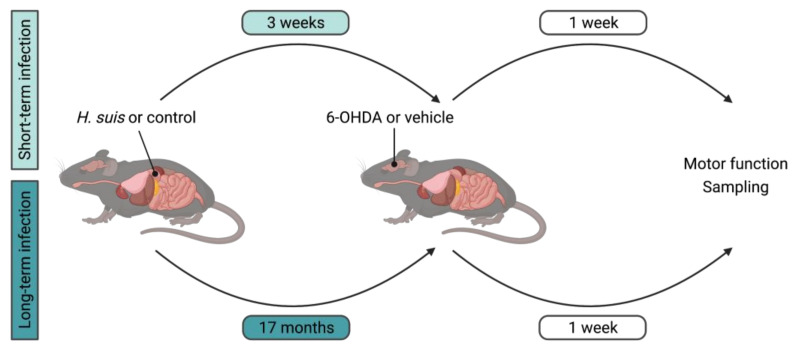
Experimental set-up to investigate the effect of *Helicobacter suis* (*H. suis*) infection in a 6-hydroxydopamine (6-OHDA) mouse model of Parkinson’s disease (PD). Animal experiments were carried out consisting of a short- and long-term infection group, respectively. C57BL/6OlaHsd mice were intragastrically inoculated at the age of 42 days with 300 µL of a stock solution containing 1 × 10^8^ viable *H. suis* HS1 bacteria/mL (n = 10 for short-term and n = 11 for long-term) or with the growth medium of the bacteria (i.e., *Brucella* broth (pH 5)) as negative controls (n = 10 for short-term and n = 9 for long-term). Twenty-one days post-infection (short-term group) and 500 days post-infection (long-term group), mice were injected in the left striatum with either 1 µL of 6-OHDA hydrochloride solution (16 µg/µL 6-OHDA in 0.2% ascorbic acid) or the vehicle solution (0.2% ascorbic acid in phosphate buffered saline). Seven days after intrastriatal injection, mice were sampled. Behavior and motor function tests were performed at two different timepoints. Baseline testing took place in the week before intrastriatal injection. The second testing took place 5–6 days after intrastriatal injection and 1–2 days prior to sampling.

**Figure 2:**
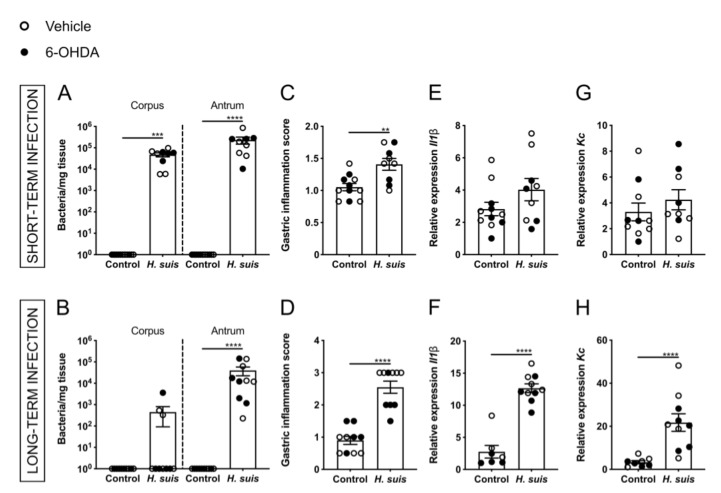
Both short- and long-term gastric *Helicobacter suis* (*H. suis*) infection is associated with gastric colonization and inflammation. (**A**,**B**) *H. suis* colonization in the stomach (corpus and antrum) of mice infected with *H. suis* or the control broth for a short- (n = 9–11) (**A**) and long-term period (n = 10) (**B**), 7 days after instrastriatal injection with either 6-OHDA (black) or vehicle (white). (**C**,**D**) Gastric inflammation score according to the Updated Sydney System based on haematoxylin and eosin (H&E) staining of the stomach of control and *H. suis*-infected mice for short- (n = 9–11) (**C**) and long-term infection (n = 10) (**D**). (**E**–**H**) Relative mRNA gene expression of the cytokine interleukin 1β (*Il1β*) and the chemokine keratinocyte chemoattractant (*Kc*) in the corpus of the stomach after short- (n = 9–11) (**E**) and long-term infection (n= 7–10) (**F**). Data were analyzed by the Mann–Whitney test. ** 0.001 ≤ *p* < 0.01; *** 0.0001 ≤ *p* < 0.001; **** *p* < 0.0001.

The experimental set-up is depicted in Figure 1. Colonization of *H. suis* in the corpus and antrum of the stomach was confirmed after sampling, in both the short- and long-term infected mice (Figure 2A,B). In the short-term group, the corpus and antrum of the stomach of *H. suis-*infected animals were colonized by an average of 4.81 × 10^4^ and 2.33 × 10^5^ bacteria per mg tissue, respectively. In the long-term group, the corpus and the antrum were colonized by an average of 4.49 × 10^2^ and 4.02 × 10^4^ bacteria per mg tissue, respectively. No *H. suis* DNA was detected in the stomach of control mice intragastrically inoculated with sterile culture medium (Figure 2A,B). Analysis of H&E-stained stomach showed infiltration of mononuclear and polymorphonuclear cells in the mucosa and submucosa of *H. suis*-infected mice (Supplementary Figure S1), indicating gastric inflammation as scored by the Updated Sydney System (Figure 2C,D) was more pronounced in long-term compared to short-term infection. Control animals of both groups, intragastrically inoculated with sterile culture medium, showed no or negligible infiltration of inflammatory cells. *H. suis*-infected animals also displayed increased expression of pro-inflammatory cytokines *Il1β* and *Kc* in the corpus of the stomach, which was significant in case of long-term infection (Figure 2E–H), while no changes in expression levels of other inflammatory markers (i.e., *Il6*, *Lix*, *Il10*, *Il17a,* and *Tnf*) were demonstrated (data not shown). We observed no significant changes in expression levels of inflammatory genes that were tested in the antrum of the stomach in the infected mice (data not shown).

### 2.2. Gastric H. suis Infection Is Associated with A Limited Decrease in Integrity of the Gastrointestinal Barrier

**Figure 3:**
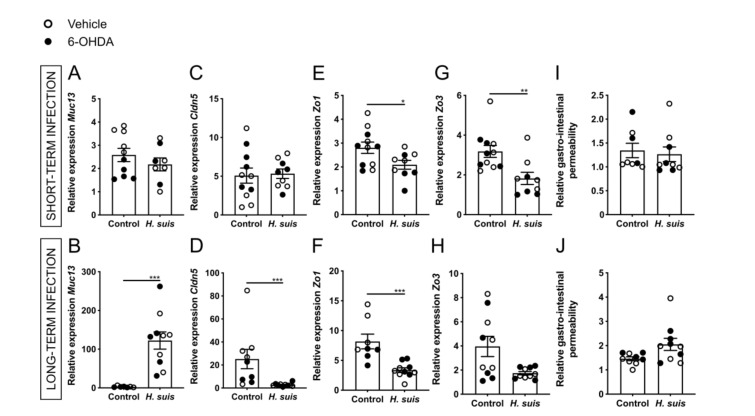
Gastric *Helicobacter suis* (*H. suis*) infection is associated with a limited decrease in integrity of the gastrointestinal barrier. (**A**,**B**) Relative mRNA gene expression of the mucin *Muc13* in the corpus of the stomach of mice infected with *H. suis* or the control broth for a short- (n = 8–10) (**A**) or long-term period (n = 7–10) (**B**), 7 days after intrastriatal injection with either 6-OHDA (black) or vehicle (white). (**C**–**H**) Relative expression of the tight junction genes claudin 5 (*Cldn5*) (**C**,**D**), zonula occludens 1 (*Zo1*) (**E**,**F**), and zonula occludens 3 (*Zo3*) (**G**,**H**) in the corpus of the stomach of mice infected with *H. suis* or the control broth for a short- (n = 9–11) (**C**,**E**,**G**) or long-term period (n = 8–10) (**D**,**F**,**H**). (**I**,**J**) Relative gastrointestinal permeability based on the 4 kDa FITC-dextran leakage assay of control mice compared to mice infected with *H. suis* for a short- (n = 8–9) (**I**) or long-term period (n = 9–10) (**J**). Data were analyzed by the Mann–Whitney test. * 0.01 ≤ *p* < 0.05; ** 0.001 ≤ *p* < 0.01; *** 0.0001 ≤ *p* < 0.001.

The gastrointestinal barrier is composed of a mucus layer overlaying a monolayer of tightly connected epithelial cells [39,40]. The mucus layer consists of a secreted mucosal barrier and an adherent mucosal barrier that protect the epithelial cells against chemical, enzymatic, microbial, and mechanical insults [39]. Aberrant expression of transmembrane mucins, such as *Muc13*, might contribute to loss of epithelial cell polarity and cell–cell interactions [41,42]. Gene expression analysis revealed a significantly increased expression of *Muc13* in the stomach corpus in the long-term but not in the short-term infection group (Figure 3A,B). Expression levels of mucins (i.e., *Muc1*, *Muc5ac*, and *Muc6*) in the stomach were not significantly altered (data not shown). Tight junctions, consisting of both transmembrane and cytosolic proteins, are crucial elements for maintaining cell-to-cell adhesion of the epithelial cells underneath this mucus layer [43]. The expression levels of the tight junction genes claudin 5 (*Cldn5*) and zonula occludens 1 (*Zo1*) were significantly decreased in the corpus of long-term infected mice (Figure 3D,F). In short-term infected mice, a significant decrease of *Zo1* and *Zo3* was demonstrated in the corpus (Figure 3E,G). No significant changes in expression levels of occludin (*Ocln*), claudin 1 (*Cldn1*), and claudin 3 (*Cldn3*) were seen (data not shown). In both groups there were no significant changes in tight junction expression levels in the antrum of the stomach (data not shown). The gastrointestinal barrier integrity was also assessed by measuring the level of fluorescence in the plasma 5 h after intragastric FITC-dextran gavage. While a small increase in fluorescence was seen in the plasma of long-term *H. suis*-infected mice, no significant changes were demonstrated (Figure 3I,J). An increased gastrointestinal barrier permeability might lead to leakage of inflammatory cytokines, chemokines, bacterial components, or even *H. suis* bacteria themselves into the bloodstream, thereby inducing systemic inflammation [44]. Therefore, the level of cytokines and chemokines in the plasma or serum was determined using a Bio-Plex assay, however, no significant changes could be demonstrated for IL-1β, IL-6, KC, IL-10, and IL-17a (data not shown).

### 2.3. H. suis-Infected Mice Are Partially Protected against the Motor Deficits Induced by Intrastriatal 6-OHDA Injection

Since *H. suis* DNA was found more often in patients with idiopathic PD [28], *H. suis* infection was shown to affect the brain in a mouse study [32], and because *H. suis* eradication resulted in improvement of motor symptoms in a PD patient [29], we hypothesized that *H. suis* might worsen motor function in PD. In all four behavior and motor function tests, no significant differences between the performance of the mice of different subgroups were demonstrated at baseline prior to intrastriatal injection (Supplementary Figure S2), indicating that the gastric *H. suis* infection alone did not result in behavior and/or motor function alterations.

**Figure 4:**
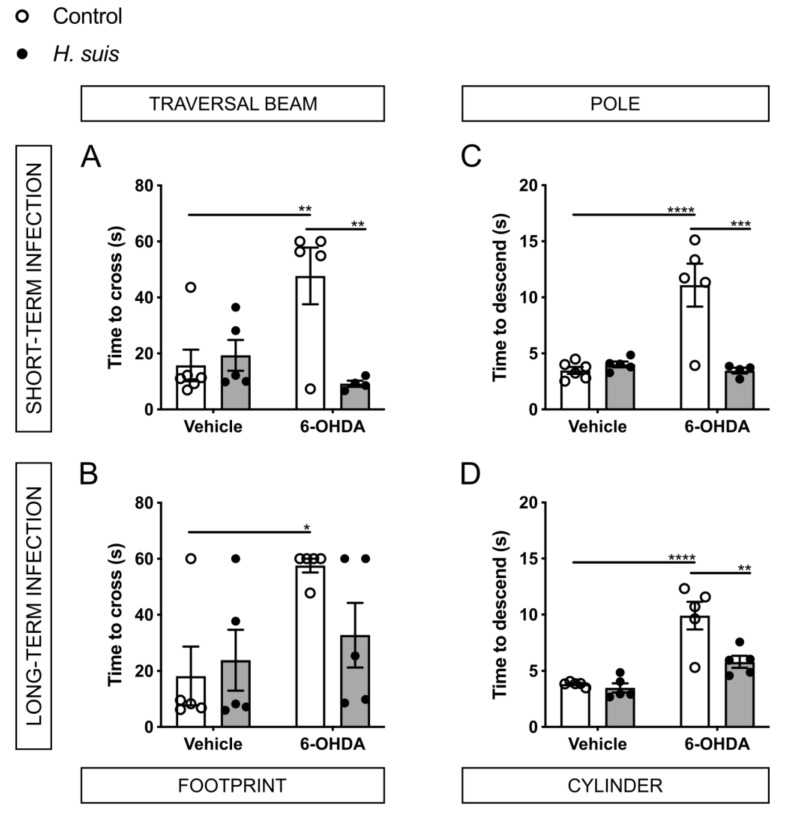

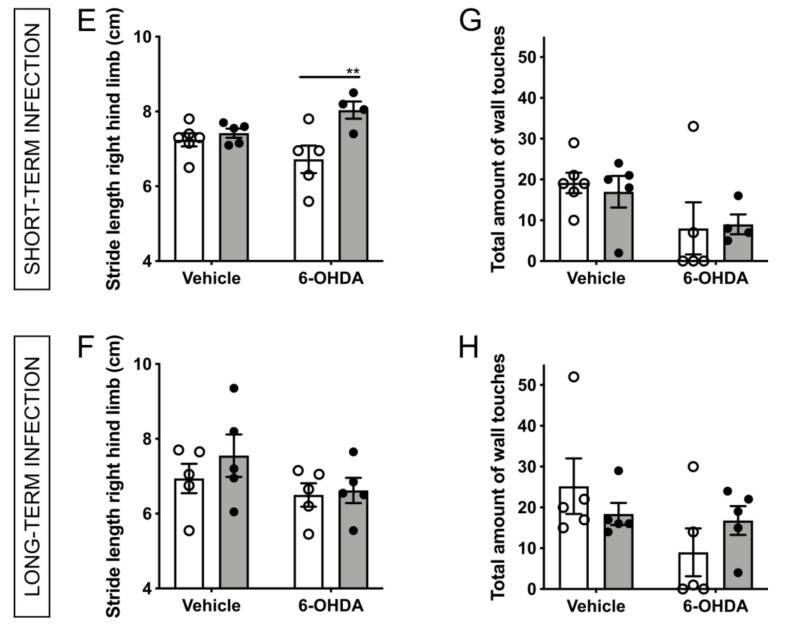
*Helicobacter suis* (*H. suis*)*-*infected mice are partially protected against the motor deficits induced by intrastriatal 6-hydroxydopamine (6-OHDA) injection. (**A**,**B**) Time (in seconds (s)) to cross the traversal beam, 7 days after intrastriatal injection with either 6-OHDA (n = 4–5) or vehicle (n = 5–6), of mice infected with *H. suis* (black) or the control broth (white) for a short-term (**A**) or long-term period (**B**). (**C**,**D**) Time (in seconds (s)) to descend the pole, 7 days after intrastriatal injection with either 6-OHDA (n = 4–5) or vehicle (n = 5–6), of mice infected with *H. suis* (black) or the control broth (white) for a short- (**C**) or long-term period (**D**). (**E**,**F**) Stride length of the right hind limb according to the footprint analysis, 7 days after intrastriatal injection with either 6-OHDA (n = 4–5) or vehicle (n = 5–6), of mice infected with *H. suis* (black) or the control broth (white) for a short-term period (**E**) or a long-term period (**F**). (**G**,**H**) Total amount of wall touches in the cylinder test, 7 days after intrastriatal injection with either 6-OHDA (n = 4–5) or vehicle (n = 5–6), of mice infected with *H. suis* (black) or the control broth (white) for a short- (**A**) or long-term period (**B**). * 0.01 ≤ *p* < 0.05; ** 0.001 ≤ *p* < 0.01; *** 0.0001 ≤ *p* < 0.001; **** *p* < 0.0001.

In the traversal beam test of both groups at 7 days post-injection in the left striatum (7 dpi) (Figure 4A,B), the control/6-OHDA subgroup needed more time to cross the beam compared to the other three subgroups. This was significant compared to the control/vehicle subgroup for both the short- and long-term infection group and compared to the *H. suis*/6-OHDA subgroup for the short-term infection group. Surprisingly, the *H. suis*/6-OHDA subgroup performed similar to both vehicle subgroups.

Similarly, in the pole test of both groups at 7 dpi (Figure 4C,D), the control/6-OHDA subgroups needed more time to descend the pole compared to the other three subgroups, which was significant between the control/vehicle and control/6-OHDA subgroups and the *H. suis*/6-OHDA and control/6-OHDA subgroups.

PD patients tend to show a shuffling gait pattern with a shortened stride length [45]. Therefore, the stride length of the mice was analyzed using the footprint analysis (Figure 4E,F). The short-term group showed a significantly shorter stride length of the control/6-OHDA subgroup compared to the *H. suis*/6-OHDA subgroup. For the long-term group, the stride length of both the control/6-OHDA and *H. suis*/6-OHDA subgroups were shorter compared to the other subgroups as well, albeit not significantly.

In the cylinder test at 7 dpi of the short-term group (Figure 4G), the total amount of wall touches (i.e., sum of left paw wall touches, right paw wall touches, and both paw wall touches) was lower for the control/6-OHDA and the *H. suis*/6-OHDA subgroup compared to the other subgroups, albeit not significantly. For the long-term group at 7 dpi (Figure 4H), the total amount of wall touches was less for the control/6-OHDA compared to the three other subgroups, albeit not significantly. Due to the unilateral left intrastriatal injection, we expected a higher number of left paw wall touches, however, no significant differences were observed (data not shown).

### 2.4. H. suis-Infected Mice Are Partially Protected against the Loss of Dopaminergic Neurons Induced by Intrastriatal 6-OHDA Injection

**Figure 5:**
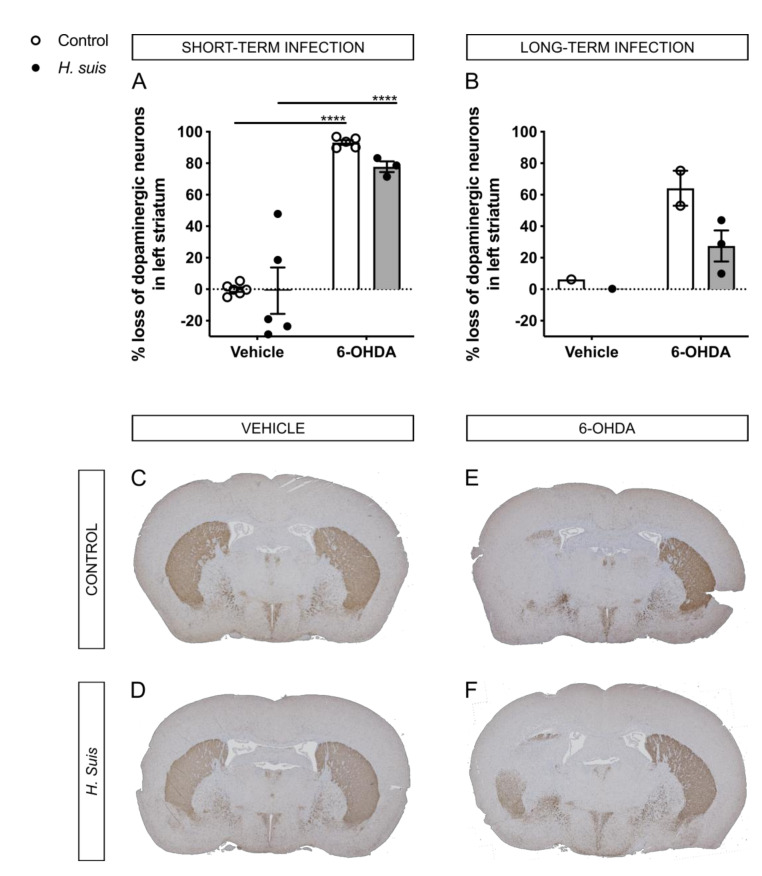
*Helicobacter suis* (*H. suis*)-infected mice are partially protected against the loss of dopaminergic neurons induced by intrastriatal 6-hydroxydopamine (6-OHDA) injection. (**A**,**B**) Loss of dopaminergic neuron fiber density in the left compared to the right striatum based on tyrosine hydroxylase (TH) staining, 7 days after intrastriatal injection with either 6-OHDA (n = 2–5) or vehicle (n = 1–6), of mice infected with *H. suis* (black) or the control broth (white) for a short- (**A**) or long-term period (**B**). (**C**–**F**) Representative whole-brain section images of the tyrosine hydroxylase staining (brown) for dopaminergic neuron fiber density in the striatum of mice from a control/vehicle subgroup (**C**), *H. suis/*vehicle subgroup (**D**), control/6-OHDA subgroup (**E**), and *H. suis*/6-OHDA subgroup (**F**). **** *p* < 0.0001.

Via TH-immunochemistry, the dopaminergic neuron fiber density was visualized in brain sections containing the striatum. The TH signal (% brown staining) of the right, non-injected striatum was set at 100% (i.e., no loss of dopaminergic neurons), and the TH signal in the left, injected striatum was then calculated with the conversion factor. For the short-term group, the mean % loss of dopaminergic neuron fiber density in the left striatum compared to the right striatum was 93.17% for the control/6-OHDA subgroup, and 77.78% for the *H. suis*/6-OHDA subgroup (Figure 5A). As expected, no loss was demonstrated in both vehicle-injected subgroups. For the long-term group, fewer data was available due to insufficient quality of brain slices usable for TH staining. Still, based on the available data, the mean % loss of dopaminergic neuron fiber density in the left striatum compared to the right striatum was 64.16% for the control/6-OHDA subgroup, and 27.49% for the *H. suis*/6-OHDA subgroup (Figure 5B). In Figure 5C–F, representative images for TH staining of all subgroups are shown.

### 2.5. 6-OHDA-Induced Gliosis Is not Influenced by H. suis Infection

**Figure 6:**
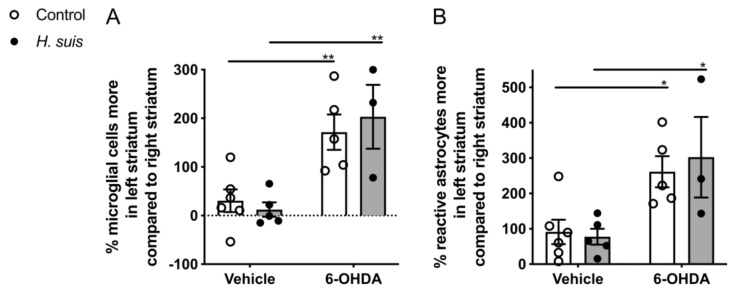
6-hydroxydopamine (6-OHDA) induced gliosis is not influenced by short-term *Helicobacter suis* (*H. suis*) infection. (**A**) Percentage (%) of IBA1-positive microglial cells more in the left injected striatum compared to the right non-injected striatum based on manual analysis, 7 days after intrastriatal injection with either 6-OHDA (n = 3–5) or vehicle (n = 5–6), of mice infected with *H. suis* (black) or the control broth (white) for a short-term period. (**B**) Percentage (%) of GFAP-positive astrocytes more in the left injected striatum compared to the right non-injected striatum based on automated analysis, 7 days after intrastriatal injection with either 6-OHDA (n = 3–5) or vehicle (n = 5–6), of mice infected with *H. suis* (black) or the control broth (white) for a short-term period. * 0.01 ≤ *p* < 0.05; ** 0.001 ≤ *p* < 0.01.

Brain sections were stained for IBA1, a marker for microglia, which are central mediators of neuroinflammation. Similar to the TH staining, the % IBA1-positive microglial cells in the injected striatum was calculated by comparison to the non-injected striatum which was set at 100% (i.e., normal number of microglial cells), and this was analyzed by manual counting (Figure 6A) for the short-term group. The percentage IBA1-positive microglial cells in the left striatum compared to the right striatum was clearly higher for the control/6-OHDA and the *H. suis*/6-OHDA subgroups, and this was significant between the *H. suis*/6-OHDA and the *H. suis*/vehicle subgroups, as well as between the control/6-OHDA and control/vehicle subgroup. The number of activated microglial cells was similar in the left and right striatum of both vehicle-injected subgroups. We also performed an immunostaining to visualize reactive astrocytes using GFAP, which was automatically analyzed and calculated in a similar way as the TH and the IBA1 staining (Figure 6B). For all subgroups, the % of reactive astrocytes was higher in the left, injected striatum compared to the right, non-injected striatum. In addition, this % was significantly higher for the control/6-OHDA compared to the control/vehicle subgroup, and for the *H. suis*/6-OHDA compared to the *H. suis*/vehicle subgroup. In Supplementary Figure S3, representative images for IBA1 and GFAP staining of all subgroups are shown.

### 2.6. Changes in Oxidative Stress Pathways in the CNS could Explain the Neuroprotective Effect seen in H. suis-Infected Mice

The loss of dopaminergic neurons in the substantia nigra and the intraneuronal aggregation of alpha-synuclein are the main pathological hallmarks of PD, which are both mechanistically linked to oxidative stress [46,47]. For this reason, we hypothesized that expression changes in genes related to oxidative stress pathways could explain the differences we see for dopaminergic cell death and motor symptoms. More specifically, we focused on the important central regulator of cellular oxidative stress responses, nuclear factor (erythroid-derived 2)-like 2 (*Nrf2*), which has also been specifically implicated in PD pathogenesis [48,49,50]. In addition, we investigated the gene expression of endogenous peroxidases, acting as antioxidant enzymes through their removal of ROS. The 6-OHDA model is ideal to investigate oxidative stress in PD, due to its main mechanism of action involving intra- or extracellular auto-oxidation leading to the production of ROS such as hydrogen peroxide and superoxide and hydroxyl radicals, further leading to oxidative stress and mitochondrial dysfunction and ultimately cell death [35]. 

**Figure 7:**
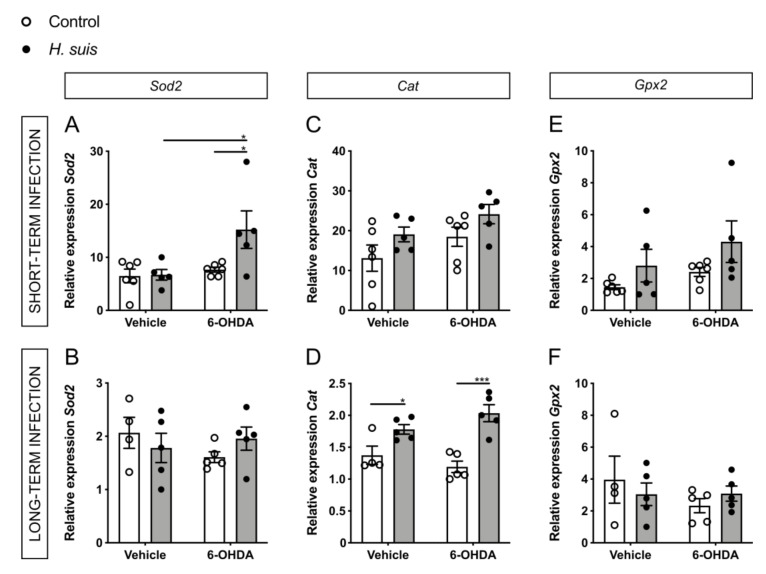
*Helicobacter suis* (*H. suis*) infection is associated with an increased gene expression of endogenous peroxidases. (**A**–**F**) Relative mRNA gene expression of the endogenous peroxidases super oxide dismutase 2 (*Sod2*) (**A**,**B**), catalase (*Cat*) (**C**,**D**), and glutathione peroxidase 2 (*Gpx2*) (**E**,**F**) in the forebrain, 7 days after intrastriatal injection with either 6-hydroxydopamine (6-OHDA) (n = 5) or vehicle (n = 4–5), of mice infected with *H. suis* (black) or the control broth (white) for a short-term period (**A**,**C**,**E**) or a long-term period (**B**,**D**,**F**). * 0.01 ≤ *p* < 0.05; *** 0.0001 ≤ *p* < 0.001.

Concerning the endogenous peroxidases, superoxide dismutases (*Sod1* and *Sod2*) convert superoxide radicals to form H_2_O_2_, which is subsequently detoxified by catalase (*Cat*) and glutathione peroxidase (*Gpx1* and *Gpx2*) [51]. The expression levels of *Sod2* showed a significant increase for the 6-OHDA-injected *H. suis* short-term infected group with a similar trend in the long-term group (Figure 7A,B), no significant changes were seen for *Sod1* (Supplementary Figure S4A,B). Mice that were infected long-term with *H. suis* showed a significant increase in expression of *Cat*, both for vehicle-injected mice and 6-OHDA-injected mice (Figure 7D). This trend for increased *Cat* expression, although not significant, was also seen in mice with a *H. suis* short-term infection (Figure 7C). When comparing the 6-OHDA-injected groups, there was no significant difference for *Gpx1* (Supplementary Figure S4C,D) and *Gpx2* (Figure 7E,F) between *H. suis*-infected mice and controls, albeit there is a trend towards the same differences seen for *Sod2* and *Cat.*

**Figure 8:**
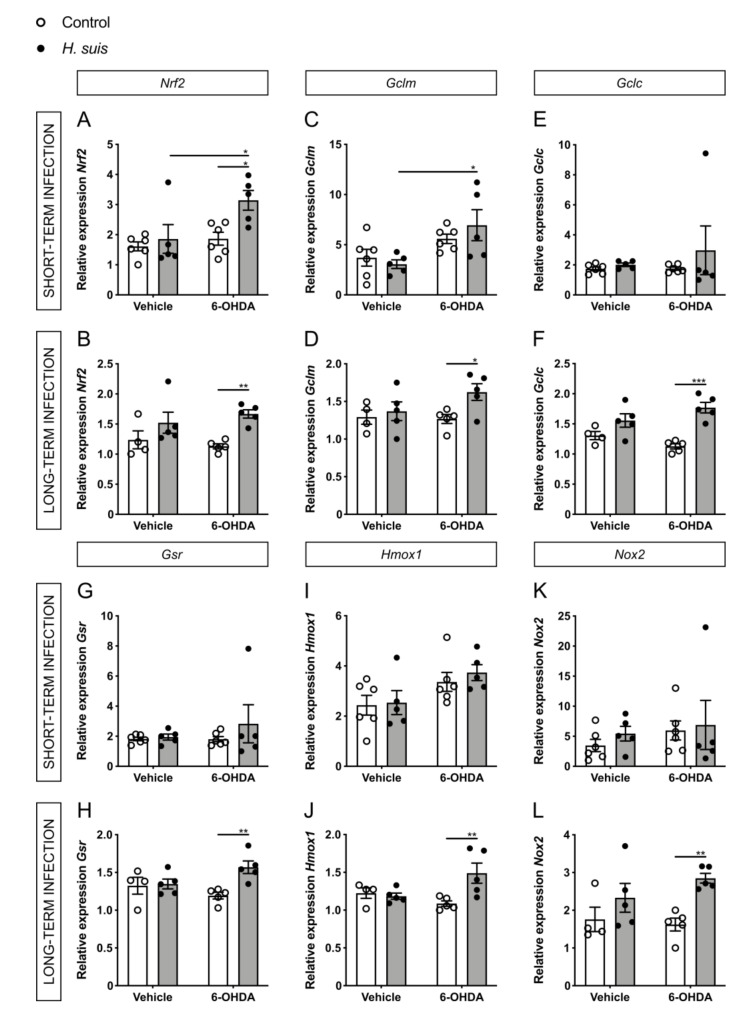
*Helicobacter suis* (*H. suis*) infection is associated with an increased gene expression of antioxidant nuclear factor (erythroid-derived 2)-like 2 (*Nrf2*)-associated genes. (**A**–**L**) Relative mRNA gene expression of *Nrf2* (**A**,**B**) and its downstream regulators glutamate-cysteine ligase modifier subunit (Gclm) (**C**,**D**), glutamate-cysteine ligase catalytic subunit (*Gclc*) (**E**,**F**), glutathi-one-disulfide reductase (*Gsr*) (**G**,**H**), heme oxygenase 1 (*Hmox1*) (**I**,**J**), and NADPH oxidase 2 (*Nox2*) (**K**,**L**) in the forebrain, 7 days after intrastriatal injection with either 6-hydroxydopamine (6-OHDA) (n = 5) or vehicle (n = 4–5), of mice infected with *H. suis* (black) or the control broth (white) for a short-term period (**A**,**C**,**E**,**G**,**I**,**K**) or a long-term period (**B**,**D**,**F**,**H**,**J**,**L**). * 0.01 ≤ *p* < 0.05; ** 0.001 ≤ *p* < 0.01; *** 0.0001 ≤ *p* < 0.001.

Fitting with our hypothesis for 6-OHDA-injected mice, the expression levels of *Nrf2* were significantly increased in the *H. suis*-infected mice compared to controls, both in the short- and long-term infected groups (Figure 8A,B). Transcriptional activation of *Nrf2* promotes cellular defense mechanisms through downstream regulators such as heme oxygenase 1 (*Hmox1*), NADPH oxidases (*Nox1* and *Nox2*), thioredoxins (*Txnrd1*, *Txn2*), glutathione synthesizing enzymes such as glutathione-disulfide reductase (*Gsr*) and glutamate-cysteine ligase (consisting of two subunits, a heavy catalytic subunit (*Gclc*) and a light regulatory subunit (*Gclm*)) with glutathione being the principal redox buffer in tissues. For the long-term infected mice, we observed a significant increase in gene expression for *Gclc*, *Gclm*, *Gsr*, *Hmox1,* and *Nox2* in parallel to the changes seen in *Nrf2* when comparing the *H. suis*/6-OHDA mice with the control/6-OHDA mice (Figure 8D,F,H,J,L). Other investigated *Nrf2*-related genes showed no significant differences, except *Txn2* in the long-term infected group (Supplementary Figure S5). No statistically significant differences were seen for the short-term infected mice for these *Nrf2*-related genes (Figure 8 C,E,G,I,K).

## 3. Discussion

While accumulating evidence points at a possible role of *H. suis* in PD, the underlying mechanism by which a gastric *H. suis* infection has an impact on the course of PD is currently unclear [28,29,52,53]. Bacterial infections, such as gastric *H. suis* infection, and its associated gastrointestinal inflammation could have their effects as trigger, facilitator, and/or aggravator [4].

Current literature tends to show exacerbating effects of *H. pylori* in PD [15] and a case report describes improvement of both the gastric and neurological symptoms in a PD patient after eradication of *H. suis* [29]. Thus, we hypothesized that a gastric *H. suis* infection might aggravate PD-related symptoms, such as motor dysfunction, and possibly PD-related pathology. Therefore, we expected the *H. suis*/6-OHDA subgroup to perform worse in the motor function tests compared to the control/6-OHDA subgroup. Surprisingly, both after a short- (1 month) and long-term infection (17 months), the *H. suis*/6-OHDA subgroup performed better than the control/6-OHDA subgroup, especially in the traversal beam and the pole test.

In accordance with the motor function, the percentage loss of dopaminergic neuron fiber density in the injected versus non-injected striatum in the control/6-OHDA subgroups was larger compared to that of the *H. suis*/6-OHDA subgroups. A gastric *H. suis* infection thus seems to partially protect against 6-OHDA induced loss of dopaminergic input in the striatum. We further compared brain gliosis between the different subgroups by analyzing microglial and astrocyte activation in the striatum. While microglial activation was demonstrated to be specific for the 6-OHDA-injected striatum, astrocyte activation was demonstrated in both vehicle- and 6-OHDA-injected striata, but more pronounced in the 6-OHDA-injected striata. This suggests that the intrastriatal injection itself caused an activation of astrocytes, but not of microglia in the striatum, and that intrastriatal 6-OHDA injection clearly results in activation of both microglia and astrocytes in the injected striatum. Indeed, astroglial and microglial activation in the nigrostriatal pathway is described in PD patients [54] and has already been demonstrated in rats after intrastriatal 6-OHDA injection [55]. However, *H. suis* infection did not have an impact on the 6-OHDA-induced gliosis.

In our study, we observed a neuroprotective effect of *H. suis* infection, specifically through pathways related to oxidative stress and mitochondrial dysfunction. Oxidative stress is an important common pathway in neurodegeneration and is tightly interconnected with the pathological hallmarks of PD, namely dopaminergic cell death and aggregation of alpha-synuclein [46,47]. Dopaminergic neurons are particularly susceptible to oxidative stress due to the normal metabolism of dopamine producing high amounts of ROS. Related to alpha-synuclein aggregates, the presence of ROS increases the amount of misfolded proteins, placing more demand on the ubiquitin-proteasome system to remove them. Post-mortem brain tissue of PD patients clearly shows evidence of oxidative stress [56,57,58,59].

*H. suis* might, similar to *H. pylori*, evoke a local oxidative stress reaction whereby superoxide radicals are produced by the bacteria themselves, and ROS and inducible nitric oxide synthase (iNOS) production by gastric epithelial cells and immune cells is induced [60,61,62,63,64,65,66,67]. The presence of moderate levels of ROS activates transcription factors that increase the antioxidant potential, thus diminishing damage by oxidative stress [51]. This transcription factor activation can also occur in the brain through gut–brain communication, especially via enteric neurons–vagal nerve communication [44]. A gastric *H. suis* infection might thus result in priming of the brain for exogenous oxidative stress, thereby increasing the chances of cell survival.

Endogenous peroxidases are important antioxidant enzymes through their removal of ROS. In the brain of *H. suis*-infected mice, we demonstrated an upregulation of endogenous peroxidases which was significant for *Sod2* and *Cat*. Although the expression of *Cat* in the brain is low in steady-state, post-mortem brain tissue of PD patients shows evidence of increased expression [58]. Importantly, the protective role of both catalase and superoxide dismutase has been shown in a 6-OHDA rat model [68].

In addition, we showed an upregulation of *Nrf2* and its downstream effectors which are important regulators of cell defense mechanisms against oxidative stress. In steady-state conditions, NRF2 is quiescent in the cytosol. However, in the case of redox imbalance, NRF2 translocates to the nucleus and binds to the antioxidant response element (ARE) which is found in the promotor of several antioxidant response genes such as heme oxygenase 1 (*Hmox1*), NADPH oxidases (*Nox1* and *Nox2*), thioredoxins (*Txnrd1*, *Txn2*), glutathione synthesizing enzymes such as glutathione-disulfide reductase (*Gsr*) and glutamate-cysteine ligase (*Gclc* and *Gclm)*. *Nrf2* has been implicated in key determinants of PD pathogenesis, namely mitochondrial impairment, oxidative stress and synucleinopathy. Multiple PD animal models have already shown the importance of the *Nrf2* pathway with overexpression protecting against cell loss and alpha-synuclein aggregation, while *Nrf2* loss exacerbates neurodegeneration [69,70,71,72,73,74,75,76,77]. Recently, the systemic activation of the *Nrf2* pathway was established in a human PD cohort, linking this activation to both alpha-synuclein pathology and the clinical disease progression [50]. Post-mortem brain tissue of PD patients also shows evidence of increased *Nrf2*-regulated genes [78,79,80,81]. All this accumulating data supports that *Nrf2* can influence PD pathology, which is replicated in our study in the *H. suis*-infected mice.

Importantly, there are some limitations to the current study. The 6-OHDA mouse model induces acute and pronounced dopaminergic neurodegeneration which contrasts with the slowly progressive disease in humans. In addition, the importance of oxidative stress could be magnified in this mouse model due to the inherent oxidative mechanism of action of 6-OHDA. In our current experiments, we sampled one week after 6-OHDA injection. It could be interesting to see what the effects of *H. suis* infection are in the long-term after dopaminergic cell loss. To conclude, the moment of *H. suis* infection could also be important, namely before or after the onset of disease, and could have different effects on disease progression or symptoms accordingly.

In summary, our study unexpectedly shows that a gastric *H. suis* infection protects, at least partially, against 6-OHDA-induced dopaminergic cell loss and motor function impairment in a unilateral intrastriatal 6-OHDA PD mouse model. As a potential explanation for this protective effect, we found *H. suis*-mediated changes in genes related to the reduction of oxidative stress. Specifically, brain tissue gene expression changes in the *Nrf2* pathway and endogenous peroxidases were shown to occur in parallel with the observed neuroprotective effect of *H. suis*. This study adds to the accumulating evidence that antioxidant response pathways, with an emphasis on Nrf2, are promising targets for therapeutic interventions in PD and that these can be targeted through gut bacteria.

## 4. Materials and Methods

### 4.1. Animals

Five-week-old female C57BL/6JOlaHsd mice (n = 40) were purchased from Envigo (Envigo, Horst, the Netherlands) and housed in a conventional animal facility. The animals were kept in filtertop cages with a 12 h light/12 h dark cycle. They had free access to an autoclaved commercial diet (2018 GLOBAL, containing 18% protein; Envigo) and water, and they were monitored daily. All animal experiments were approved by the Ethical Committee of the Faculty of Veterinary Medicine, Ghent University, Belgium (EC2016/21 and EC2017/17).

### 4.2. Cultivation of H. suis and In Vivo Infection Procedure

The porcine *H. suis* isolate HS1 was originally obtained according to the method previously described [82]. The bacteria were grown under microaerobic conditions (85% N_2_, 10% CO_2_, 5% O_2_) at 37°C on biphasic *Brucella* culture plates (Becton-Dickinson, Erembodegem, Belgium) supplemented with 20% inactivated fetal calf serum (Hyclone, Thermo Fisher Scientific, Waltham, MA, USA), 5 mg/L amphotericin B (Sigma-Aldrich, Saint Louis, Missouri, USA), *Campylobacter* selective supplement (Skirrow, Oxoid, Basingstoke, UK; containing 10 mg/L vancomycin, 5 mg/L trimethoprim lactate, and 2500 U/L Polymyxin B), and Vitox supplement (Oxoid). *Brucella* broth (Oxoid) was added on top of the agar to obtain biphasic culture conditions. The pH of both agar and broth was adjusted to 5 by adding HCl to a final concentration of 0.05%. The bacteria were passaged at least twice to ensure reliable growth. After incubation, the bacteria were harvested and the number of viable (motile) bacteria/mL was microscopically determined using an improved Neubauer counting chamber (Sigma-Aldrich). The bacteria were diluted to a final concentration of 1 × 10^8^ viable bacteria/mL.

At the age of 6 weeks, 10 (short-term infection) or 11 (long-term infection) mice were intragastrically inoculated with 300 µL of a stock solution containing 1 × 10^8^ viable *H. suis* HS1 bacteria/mL. The remaining 10 (short-term infection) or 9 (long-term infection) mice served as negative controls and received the growth medium of the bacteria (i.e., *Brucella* broth (pH 5)). One month post-infection (short-term infection) and 17 months post-infection (long-term infection), mice were euthanized for further analysis by the administration of an overdose of ketamine/xylazine followed by decapitation.

### 4.3. Intrastriatal 6-OHDA Injection Procedure

One week prior to sampling, mice were placed one by one in random order in an induction chamber and anesthesia was induced with 2–2.5% isoflurane (Medini, Oostkamp, Belgium) in pure oxygen with a delivery rate of 0.9 L/min until loss of paw withdrawal reflex. After induction, mice were moved to a stereotactic apparatus (Neurostar, Tübingen, Germany) where general anesthesia was maintained with 1.5–1.8% isoflurane delivered in 100% oxygen. Anesthetic depth was assessed regularly throughout the entire procedure by monitoring respiratory rate and using the toe and tail pinch. To prevent hypothermia, mice were placed on a heated pad. The mice were fixated in the stereotactic apparatus, eye ointment was applied to prevent corneal drying, and the head was shaved and disinfected with an ethanol swab. The skull was dissected using a midline skin incision, and after identification of bregma and lambda as reference points, a hole in the skull was drilled at the injection site using the stereotactic apparatus. The freshly made 6-OHDA hydrochloride (Merck KGaA, Darmstadt, Germany) solution (16 µg/µL 6-OHDA in 0.2% ascorbic acid) or the vehicle solution (0.2% ascorbic acid in phosphate buffered saline) was then automatically drawn into the syringe. The syringe was slowly directed to the injection coordinates relative to bregma (i.e., 0.14 mm posterior, 2 mm lateral (left), and 3 mm ventral to bregma) of the left striatum and 1 µL of either the 6-OHDA solution or the vehicle solution was subsequently injected over 10 min. After injection, the needle was kept in place for another 10 min, after which the syringe was slowly retracted. Bone wax (SMI, St. Vith, Belgium) was applied to close the drill hole, the skin was sutured and both lidocaine (Xylocaine 2% gel, Medini, Oostkamp, Belgium) and iso-Betadine (iso-Betadine gel 10%, Medini, Oostkamp, Belgium) were applied. Once recovered from anesthesia, mice were placed into their home cage whereby additional warmth was provided. Daily post-operative follow-up was performed by checking the behavior, body weight, and temperature of the animals.

Eventually, both the short-term group (n = 20) and long-term group (n = 20) consisted of 4 subgroups: (1) control/vehicle, (2) control/6-OHDA, (3) *H. suis*/vehicle, and (4) *H. suis*/6-OHDA, with the first term indicating the intragastric infection and the second term indicating the left intrastriatal injection.

### 4.4. Behavior and Motor Function Tests

Behavior and motor function tests were performed at 2 different timepoints, namely at baseline and post-injection. Tests at baseline took place after intragastric inoculation and just before intrastriatal injection, i.e., 7 days pre-injection for the short-term group and 1–2 days pre-injection for the long-term group. Post-injection tests took place 5–6 days post-injection and 1–2 days prior to sampling. Four different tests (i.e., traversal beam test, pole test, footprint analysis, cylinder test) were carried out to gain insights into motor function and exploratory behavior of the mice before and after left intrastriatal 6-OHDA injection. The motor function tests were adapted from Fleming et al. [83] and were all video recorded.

For the traversal beam test, two clean mouse cages were inverted on a tabletop and a beam of 1 m length consisting of four sections with narrowing widths (3.5, 2.5, 1.5, and 0.5 cm) was placed on top of the inverted mouse cages. The home cage of the mice was placed on its side at the end of the beam so that the narrowest end of the beam led right into the home cage. The mice were picked up by the base of their tails and placed at the wide end of the beam. The time the mice needed to traverse the beam was recorded. For training trials (three or four in total per mouse), no grid was placed on the beam. For the testing trials (five in total per mouse at each timepoint), a mesh grid that corresponded to each beam width was placed on top of the beam. The mesh grid had 1 cm squares and left a 1 cm space between the grid and the beam surface. The mean of the 3 best testing trials at each timepoint was used for analysis.

For the pole test, a 50 cm long vertical pole of 1 cm in diameter was wrapped with tape (to facilitate the animals grip) and placed in a cage. Mice were placed head-down on top of the pole and time to descend into the cage was assessed. Each mouse underwent three or four training trials (immediately starting from the top of the pole), and five testing trials at each timepoint. The mean of the 3 best testing trials at each timepoint was used for analysis.

For the footprint analysis, the front and hind feet of the mice were coated with non-toxic paint of a different color. A small corridor was formed by placing two inverted mouse cages at the side of the corridor, with the home cage placed on its side at the end of this corridor. Each animal was then allowed to walk on absorbent white paper into this corridor to its home cage. After 1 training run without paint and one with paint, data were collected from 2 test runs. The stride length was then measured as the distance between the hind feet pawprints of consecutive steps. The mean stride length of the 2 test runs at each timepoint was used for analysis.

For the cylinder test, mice were placed in an open-top, clear plexiglass cylinder of 20 cm high with a diameter of 13 cm for 5 min. Forelimb activity while rearing against the wall of the cylinder was assessed, whereby only placement of the whole palm on the cylinder wall (indicative for its use for body support) was counted. Contact with either the left or right paw, as well as with both paws simultaneously was registered.

### 4.5. Collection and Processing of Samples

After sedation, blood was collected by cardiac puncture and immediately transferred to EDTA-tubes (Sarstedt, Wexford, Ireland) and centrifugated at 4 °C for 15 min at 300× *g* for plasma collection. For serum preparation, blood was collected in Eppendorfs and stored overnight at 4 °C, centrifugated twice for 15 min at 1000× *g*, after which serum was collected and stored at −70 °C. Mice were then transcardially perfused with D-Phosphate Buffered Saline (PBS)/heparin (0.2% heparin). The stomach was isolated, cut open via the greater curvature and rinsed with sterile Hank’s Buffered Salt solution (Gibco by Thermo Fisher Scientific, Waltham, MA, USA). Samples from the corpus and the antrum of the stomach were collected and stored in RNAlater (Qiagen, Hilden, Germany). A longitudinal section starting from the forestomach and comprising the corpus and antrum of the stomach and part of the duodenum was taken and fixated overnight in 10% formalin at 4 °C and embedded in paraffin for H&E staining.

For the brain sampling, striatum dissection was the priority to allow for dopaminergic cell death as an outcome parameter. To isolate the brain slice containing the injection site, a vertical cut was made at the rostral border of the hypothalamus and at 2 mm caudal from the frontal lobe. This brain slice containing the injection site was dissected and stored overnight in 10% formalin at 4 °C and embedded in paraffin. The brain part rostral of the brain slice (the forebrain, including prefrontal cortex and olfactory bulbs) was snap frozen for subsequent gene expression analysis. In the long-term group, this forebrain part did not contain the olfactory bulbs.

### 4.6. DNA Extraction and Quantification of Colonizing H. suis in the Stomach

The gastric samples that were stored in RNAlater (Qiagen, Hilden, Germany) were homogenized (Tissuelyser, Qiagen) and RNA and DNA were separated using TriReagent RT (Molecular Research Center Inc, Cincinnati, USA) according to the manufacturer’s instructions. The number of colonizing bacteria per mg tissue was determined in the DNA samples using Real-Time (RT)-quantitative (q) Polymerase Chain Reaction (PCR) specific for the detection of *H. suis* [84]. The *H. suis* standard was generated as previously described [85]. Both standard and samples were run in duplicate on a CFX384^TM^ RT-qPCR System with a C1000 Thermal Cycler (Bio-Rad, Hercules CA, USA). The Bio-Rad CFX Manager (version 1.6) software was used for the quantification of *H. suis* DNA in the tissue samples.

### 4.7. RNA Extraction and RT-qPCR for Gene Expression Analysis

Cerebral samples were first homogenized (Tissuelyser, Qiagen) and RNA and DNA were separated using Trizol (Fisher Scientific, NH, USA) according to the manufacturer’s instructions. Total RNA from cerebral tissue was further purified using the Aurum^TM^ Total RNA Mini Kit (Bio-Rad, Hercules, CA, USA), and total RNA from corpus and antrum of the stomach was further purified using the RNeasy mini kit (Qiagen) according to the manufacturer’s instructions. Purity of RNA was determined by measuring the ratio of absorbance at 260 and 280 nm with NanoDrop equipment (Nanodrop ND-1000, Thermo Fisher Scientific, Waltham, MA, USA). Next, cDNA was synthesized using the Sensifast cDNA Synthesis kit (GC Biotech) for the brain samples and the iScript^TM^ cDNA synthesis kit (Bio-Rad, Hercules, CA, USA) for the gastric samples. RT-qPCR analysis of the brain samples was performed on the Light Cycler 480 system (Roche) using the 2xSensifast SYBR No-Rox kit (GC Biotech, Waddinxveen, The Netherlands). RT-qPCR analysis of stomach samples was carried out on a CFX384^TM^ RT-qPCR System with a C1000 Thermal Cycler (Bio-Rad) using the 2xSensimix Sybr No-Rox kit (Bioline). Expression levels were normalized to the expression of reference genes as determined by the geNorm Housekeeping Gene Selection Software (i.e., *Gadph*, *Hprt*, *Rpl*, and *Ubc* for the brain samples and *Hprt, H2afz*, and *Ppia* for the stomach samples) [86]. The primer sequences of the target and housekeeping genes are shown in Table S1.

### 4.8. Quantification of the Gastrointestinal Permeability

The gastrointestinal permeability was determined as previously described [87]. Briefly, 100 µL of 25 mg/mL 4 kDa FITC-dextran (Merk KGaA, Darmstadt, Germany) were administered intragastrically 5 h before the collection of blood in EDTA-coated tubes (Sarstedt, Wexford, Ireland) by means of cardiac puncture. Following centrifugation, plasma was isolated and diluted twice in sterile D-PBS. Gastrointestinal leakage was determined by measurement of fluorescence at λ_ex_/λ_em_ = 485/520 nm using the FLUOstar Omega plate reader (BMG Labtech) and relative gastrointestinal permeability was calculated (relative to the minimum value).

### 4.9. Cytokine/Chemokine Measurements

The levels of interleukin (IL-)1β, IL-6, the murine IL-8 homolog keratinocyte chemoattractant (KC), IL-10, and IL-17a in plasma (short-term infection group) and serum (long-term infection group) were measured using the Bio-Plex cytokine assay (Bio-Rad) according to the manufacturer’s instructions. 

### 4.10. Histopathology and Immunohistochemistry

To evaluate gastric inflammation, paraffin sections of 5 µm were cut, deparaffinized and rehydrated. Sections were then stained with Hematoxylin Gill III Prosan (Merck) and Eosin Yellow (VWR, Radnor, PA, USA) according to the standardized protocols. The hematoxylin and eosin (H&E) staining was applied to determine the intensity of the overall gastritis, using the modified visual analog scale similar to the adapted Updated Sydney System as previously described [88].

To evaluate brain tissue, paraffin sections of 5 µm were cut, deparaffinized and rehydrated. To allow for comparison between the different subgroups, similar regions in the striatum were stained for each antibody, as depicted in Figure 5 and Supplementary Figure S3. For immunohistochemical staining of ionized calcium binding adapter molecule 1 (IBA1), first heat-induced antigen retrieval was performed in citrate buffer (10 mM, pH 6) and non-specific reactions and endogenous peroxidase activity were blocked by incubating the slides with 30% goat serum (30 min) and 3% H_2_O_2_ in methanol (5 min), respectively. Immunostaining of the brain sections was then performed as described before [89] using the anti-IBA1 antibody (Wako Chemicals, Neuss, Germany) at a dilution of 1/1000. 

For glial fibrillary acidic protein (GFAP) and tyrosine hydroxylase (TH) immunostaining, heat-induced antigen retrieval was performed using citrate buffer (Dako; S2031), followed by washing in PBS. Endogenous peroxidase activity was blocked with 3% H_2_O_2_ in methanol for 10 min at room temperature followed by washing with PBS. Samples were then blocked with 5% goat serum in antibody diluent (Dako; S2022) for 30 min at room temperature, followed by overnight incubation at 4 °C with either primary anti-GFAP antibody (Dako; Z0334, 1/) diluted in block buffer or with primary polyclonal rabbit anti-TH antibody (Milipore; AB152, 1/1000). The next day, slides were rinsed with PBS, and slides for GFAP staining were then incubated with biotinylated goat anti-rabbit secondary antibody (Dako; E0432, 1/500) for one hour at room temperature, once again rinsed with PBS and incubated for 30 min with VECTASTAIN^®^ Elite ABC system (Vector, PK6100). Slides for TH staining were then incubated with EnVision HRP anti-rabbit (Dako; K4003) for 1 h. After a final wash step with PBS, 3,3-diaminobenzidine (DAB, Agilent; K4011) was added for 5 min. Finally, a counterstaining with hematoxylin was performed, the slides were dehydrated and mounted with xylene-based mounting medium. IBA1, GFAP, and TH stainings of the brain sections were visualized using Zeiss Axio Scan.Z1 (Oberkochen, Germany) followed by automated quantification using Fiji and QuPath software. Quantification of the percentage brown color was done via color thresholding with correction for the total amount of tissue. 

### 4.11. Statistics

Statistical analysis was performed using GraphPad Prism. Data were analyzed by the two-way ANOVA test and are presented as means ± standard error of mean (SEM), unless stated otherwise. Significance levels are indicated on the graphs * 0.01 ≤ *p* < 0.05; ** 0.001 ≤ *p* < 0.01; *** 0.0001 ≤ *p* < 0.001; **** *p* < 0.0001.

## Data Availability

The data presented in this study are available in the article and the supplementary material.

## Supplementary Materials

The following are available online at https://www.mdpi.com/article/10.3390/ijms222111328/s1, Figure S1: Representative hematoxylin and eosin (H&E) images of control and *Helicobacter suis* (*H. suis*)*-*infected mice with a short- or long-term infection; Figure S2: Behavior and motor function tests at baseline; Figure S3: Representative images for GFAP and IBA1 stainings; Figure S4: *Helicobacter suis* (*H. suis*) infection and gene expression of endogenous peroxidases; Figure S5: *Helicobacter suis* (*H. suis*) infection and gene expression of antioxidant nuclear factor (erythroid-derived 2)-like 2 (*Nrf2*)-associated genes; Table S1: Overview of the RT-qPCR primer sequences used in the current study.
