# Supplementary material for: Gastric Helicobacter suis Infection Partially Protects against Neurotoxicity in A 6-OHDA Parkinson’s Disease Mouse Model
Source: Int J Mol Sci. 2021 Oct 20;22(21):11328. doi: 10.3390/ijms222111328 (PMC8582972; doi:10.3390/ijms222111328)
Supplement: Supplementary file 1 [file ijms-22-11328-s001.zip › ijms-1402556-revised si.pdf]

Supplementary Material

Gastric *Helicobacter suis* Infection Partially Protects Against Neurotoxicity in A 6-OHDA Parkinson's Disease Mouse Model

Helena Berlamont [1,†], Arnout Bruggeman [2,3,4,†], Eva Bauwens [1], Charysse Vandendriessche [2,3], Elien Clarebout [2,3], Junhua Xie [2,3], Sofie De Bruyckere [1], Griet Van Imschoot [2,3], Elien Van Wonterghem [2,3], Richard Ducatelle [1], Patrick Santens [4], Annemieke Smet [5], Freddy Haesebrouck [1,‡] and Roosmarijn E. Vandenbroucke [2,3,*,‡]

[1] Department of Pathobiology, Pharmacology and Zoological Medicine, Faculty of Veterinary Medicine, Ghent University, 9820 Merelbeke, Belgium; Helena.Berlamont@UGent.be (H.B.); Eva.Bauwens@UGent.be (E.B.); Sofie.DeBruyckere@UGent.be (S.D.B.); Freddy.Haesebrouck@UGent.be (F.H.); Richard.Ducatelle@UGent.be (R.D.)

[2] VIB Center for Inflammation Research, VIB, 9052 Ghent, Belgium; Arnout.Bruggeman@irc.VIB-UGent.be (A.B.); Charysse.Vandendriessche@irc.VIB-UGent.be (C.V.); Elien.Clarebout@irc.VIB-UGent.be (E.C.); Junhua.Xie@irc.VIB-UGent.be (J.X.); Griet.VanImschoot@irc.VIB-UGent.be (G.V.I.); Elien.VanWonterghem@irc.VIB-UGent.be (E.V.W.)

[3] Department of Biomedical Molecular Biology, Ghent University, 9052 Ghent, Belgium

[4] Department of Neurology, Ghent University Hospital, 9000 Ghent, Belgium; Patrick.Santens@ugent.be

[5] Laboratory of Experimental Medicine and Pediatrics, Faculty of Medicine and Health Sciences, University of Antwerp, 2610 Antwerp, Belgium; Annemieke.Smet@uantwerpen.be

* Correspondence: Roosmarijn.Vandenbroucke@irc.VIB-UGent.be (R.E.V.); Tel.: +32-9-3313730

† Shared first authorship.

‡ Shared senior authorship.

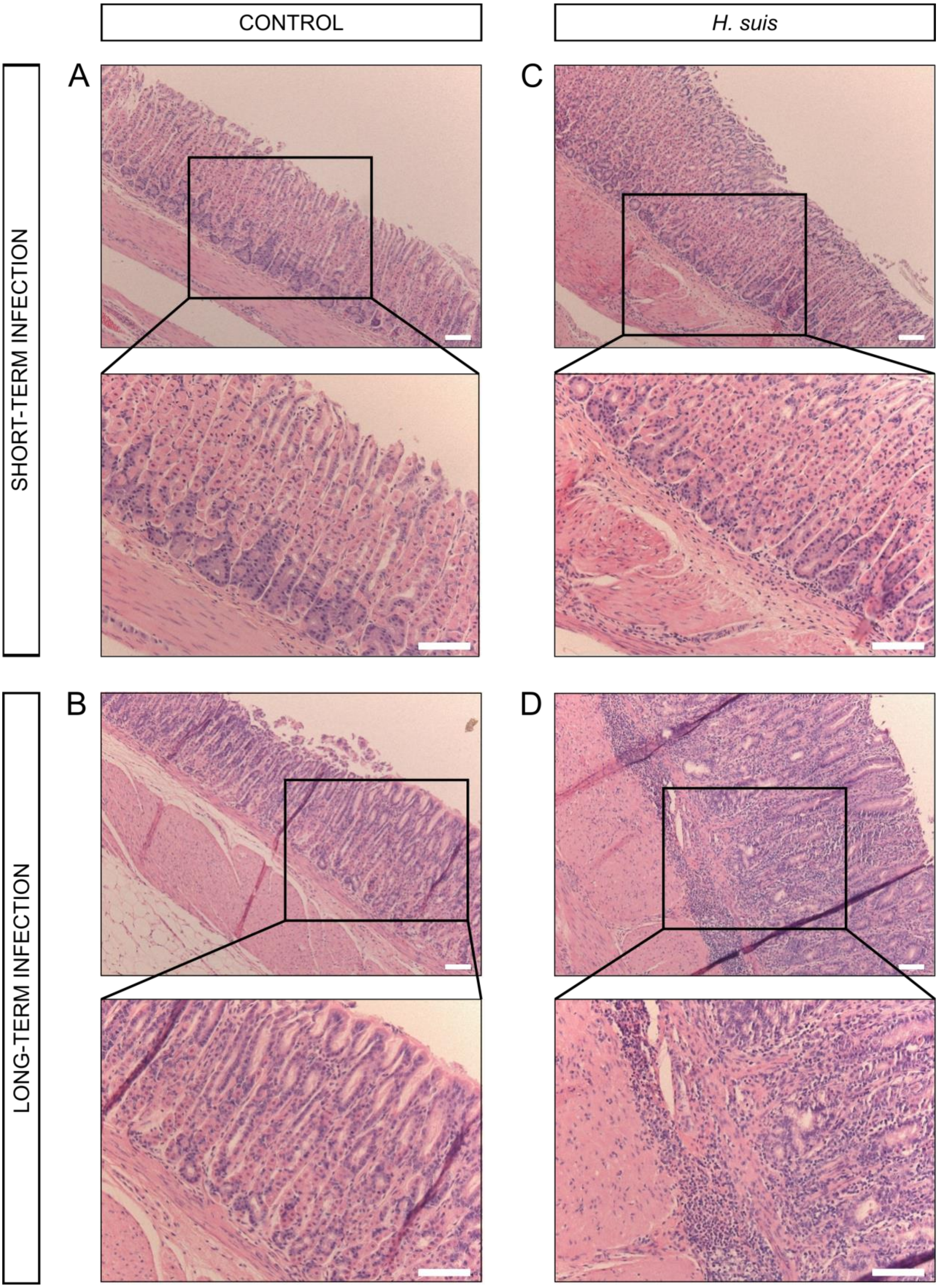

Supplementary Figure S1. Representative haematoxylin and eosin (H&E) images of control and *Helicobacter suis* (*H. suis*)-infected mice with a short- or long-term infection. (A-D) Representative images of the H&E staining of the stomach of mice infected with control broth (A, B) or *H. suis* (C, D) for a short- (A, C) or long-term period (B, D). Scale bar represents 100 µm.

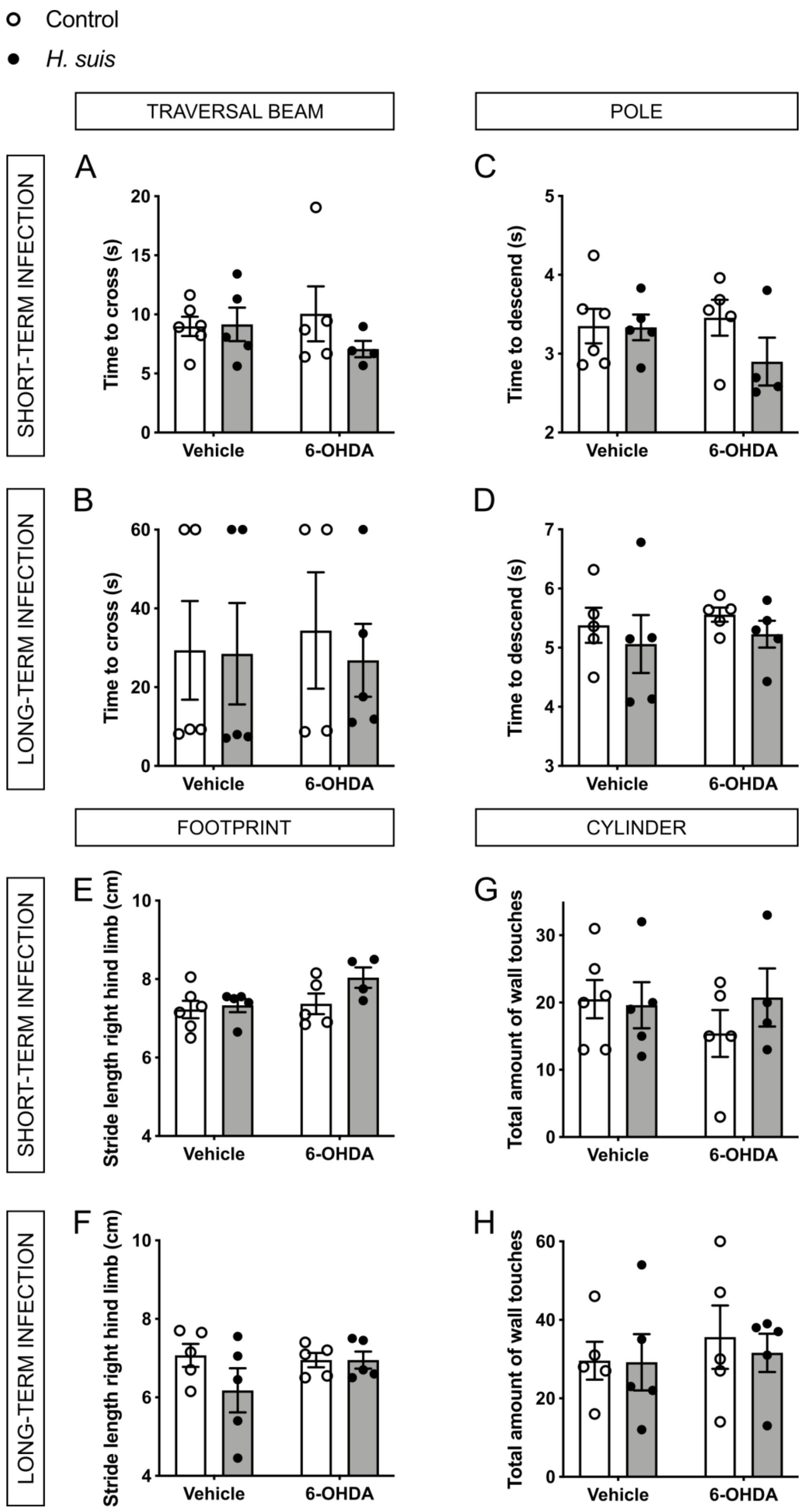

Control
H. suis
TRAVERSAL BEAM
POLE
SHORT-TERM INFECTION
LONG-TERM INFECTION
A
B
C
D
Time to cross (s)
Time to descend (s)
Vehicle
6-OHDA
FOOTPRINT
CYLINDER
E
F
G
H
Stride length right hind limb (cm)
Total amount of wall touches

Supplementary Figure S2. Behavior and motor function tests at baseline. (A-B) Time (in seconds (s)) to cross the traversal beam, in the week prior to intrastriatal injection with either 6-hydroxydopamine (6-OHDA) (n = 4-5) or vehicle (n = 5-6), of mice infected with *Helicobacter suis* (*H. suis*) (black) or the control broth (white) for a short- (A) or a long-term period (B). **(C-D)** Time (in seconds (s)) to descend the pole, in the week prior to intrastriatal injection with either 6-OHDA (n = 4-5) or vehicle (n = 5-6), of mice infected with *H. suis* (black) or the control broth (white) for a short-term period (C) or a long-term period (D). **(E-F)** Stride length of the right hind limb according to the footprint analysis, in the week prior to intrastriatal injection with either 6-OHDA (n = 4-5) or vehicle (n = 5-6), of mice infected with *H. suis* (black) or the control broth (white) for a short- (E) or long-term period (F). **(G-H)** Total amount of wall touches in the cylinder test, in the week prior to intrastriatal injection with either 6-OHDA (n = 4-5) or vehicle (n = 5-6), of mice infected with *H. suis* (black) or the control broth (white) for a short- (G) or long-term period (H).

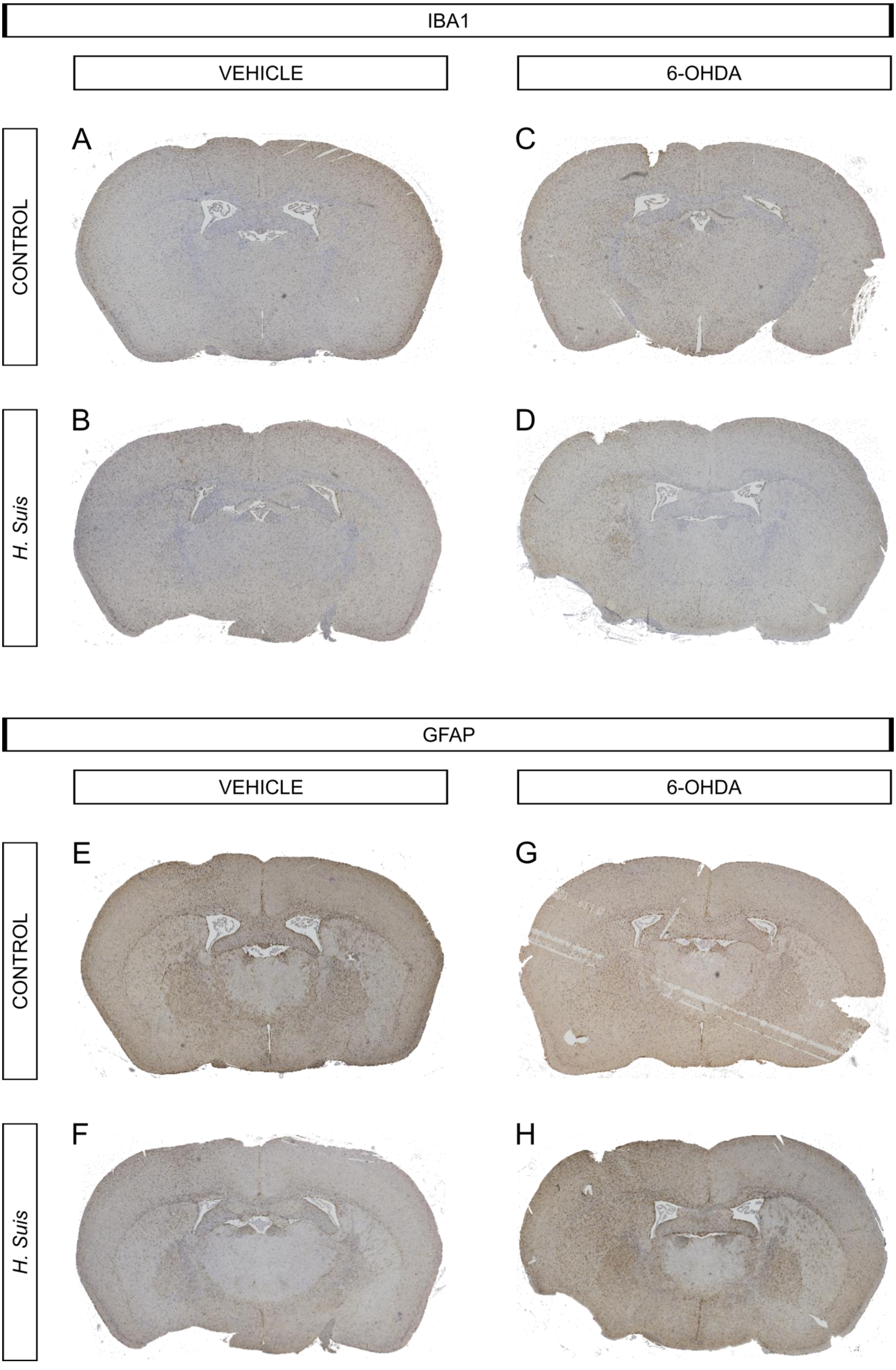
IBA1
VEHICLE
6-OHDA
CONTROL
H. Suis
A
B
C
D
GFAP
VEHICLE
6-OHDA
CONTROL
H. Suis
E
F
G
H

Supplementary Figure S3. Representative images for GFAP and IBA1 stainings. (A-D) Representative whole-brain section images of the IBA1 staining (brown) for microglia in the striatum of mice from a control/vehicle subgroup (A), *H. suis*/vehicle subgroup (B), control/6-OHDA subgroup (C) and *H. suis*/6-OHDA subgroup (D). **(E-H)** Representative whole-brain section images of the GFAP staining (brown) for astrocytes in the striatum of mice from a control/vehicle subgroup (E), *H. suis*/vehicle subgroup (F), control/6-OHDA subgroup (G) and *H. suis*/6-OHDA subgroup (H).

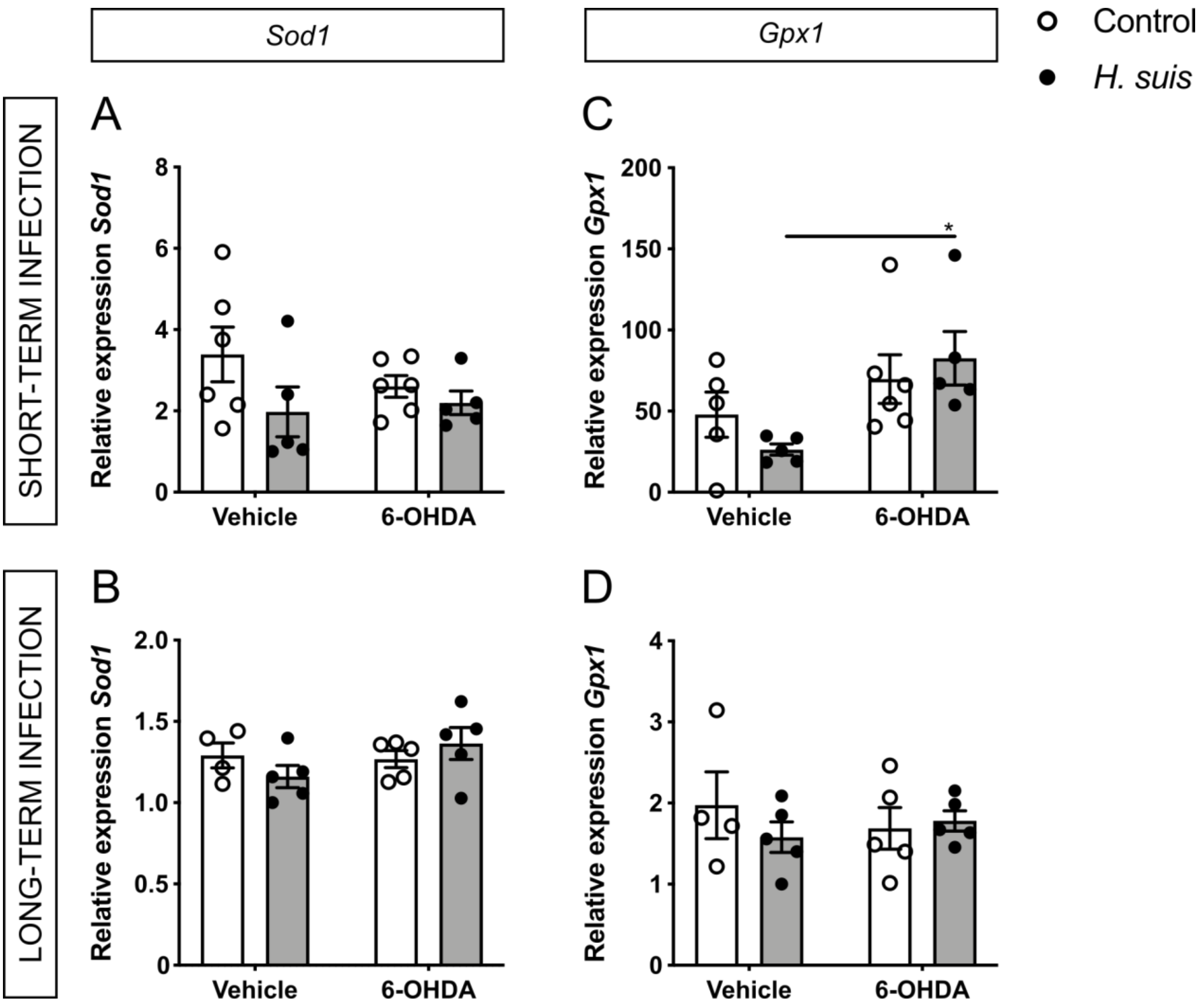

Supplementary Figure S4. *Helicobacter suis* (*H. suis*) infection and gene expression of endogenous peroxidases. (A-D) Relative mRNA gene expression of the endogenous peroxidases super oxide dismutase 1 (*Sod1*) (A-B) and glutathione peroxidase 1 (*Gpx1*) (C-D) in the forebrain, 7 days after intrastriatal injection with either 6-hydroxydopamine (6-OHDA) (n = 5) or vehicle (n = 4-5), of mice infected with *H. suis* (black) or the control broth (white) for a short- (A, C) or long-term period (B, D).

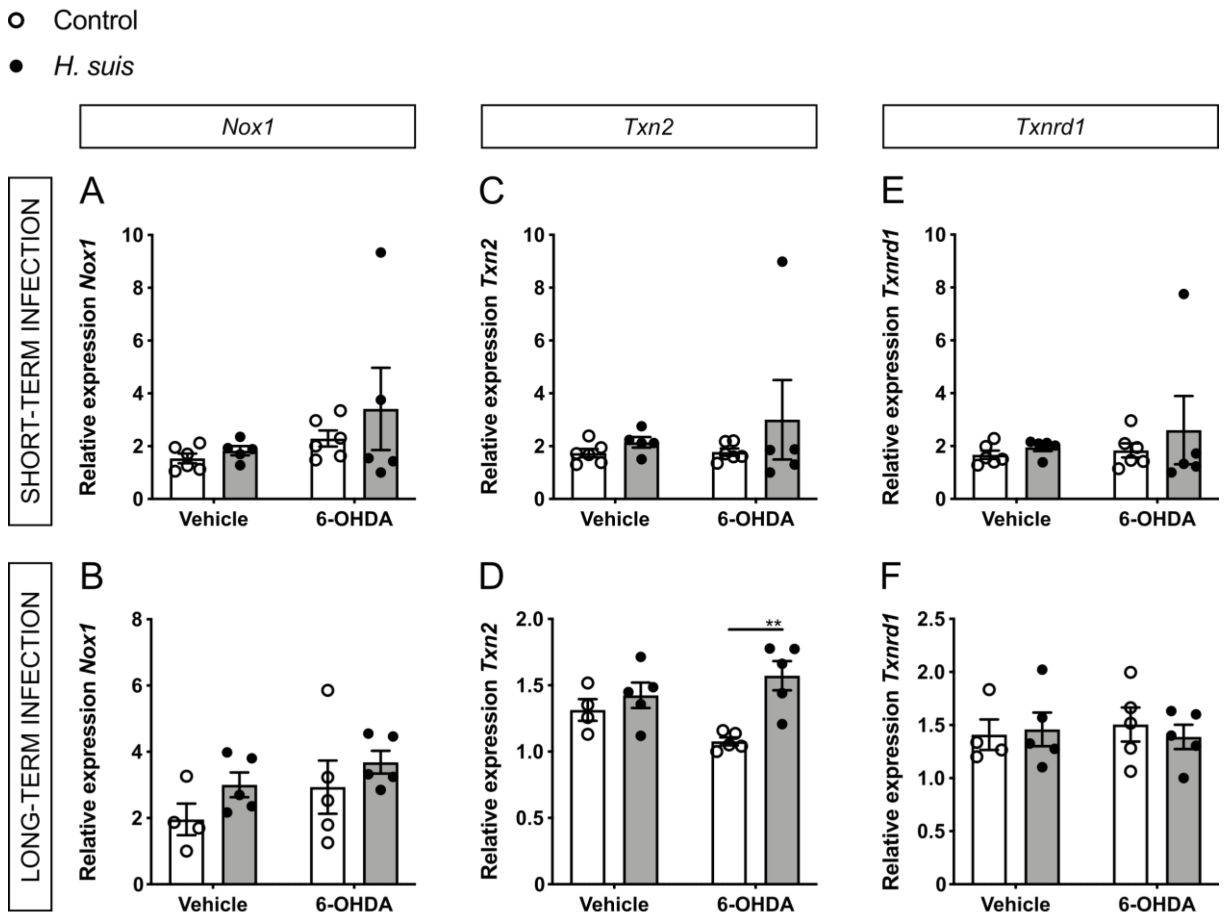

Supplementary Figure S5. ***Helicobacter suis*** **(*H. suis*) infection and gene expression of antioxidant nuclear factor (erythroid-derived 2)-like 2 (*Nrf2*)-associated genes. (A-F)** Relative mRNA gene expression of *Nrf2* downstream regulators NADPH oxidase 1 (*Nox1*) (A-B), thioredoxin 2 (*Txn2*) (C-D) and thioredoxin reductase 1 (*Txnrd1*) (E-F) in the forebrain, 7 days after intrastriatal injection with either 6-hydroxydopamine (6-OHDA) (n = 5) or vehicle (n = 4-5), of mice infected with *H. suis* (black) or the control broth (white) for a short- (A, C, E) or long-term period (B, D, F).

Table S1. Overview of RT-qPCR primer sequences used in the current study.

		Forward primer sequence	Reverse primer sequence
Stomach	*H. suis*	AAAACAMAGGCGATCGCCCTGTA	TTTCTTCGCCAGGTTCAAAGCG
	H2afz	CGTATCACCCCTCGTCACTT	TCAGCGATTTGTGGATGTGT
	Hprt	CAGGCCAGACTTTGTTGGAT	TTGCGCTCATCTTAGGCTTT
	Ppia	AGCATACAGGTCCTGGCATC	TTCACCTTCCCAAAGACCAC
	Il1β	CACCTCACAAGCAGAGCACAAG	GCATTAGAAACAGTCCAGCCCATAC
	Il6	TAGTCCTTCCTACCCCAATTTCC	TTGGTCCTTAGCCACTCCTTC
	Kc	GCTGGGATTCACCTCAAGAA	TCTCCGTTACTTGGGGACAC
	Lix	CTCAGTCATAGCCGCAACCGAGC	CCGTTCTTTCCACTGCGAGTGC
	Il10	CTGGACAACATACTGCTAACCG	GGGCATCACTTCTACCAGGTAA
	Il17a	TTTAACTCCCTTGGCGCAAAA	CTTTCCCTCCGCATTGACAC
	Tnfα	ACCCTGGTATGAGCCCATATAC	ACACCCATTCCCTTCACAGAG
	Ocln	CCAGGCAGCGTGTTCCT	TTCTAAATAACAGTCACCTGAGGGC
	Cldn1	TCTACGAGGGACTGTGGATG	TCAGATTCAGCAAGGAGTCG
	Cldn3	AAGCCGAATGGACAAAGAA	CTGGCAAGTAGCTGCAGTG
	Cldn5	GCAAGGTGTATGAATCTGTGCT	GTCAAGGTAACAAAGAGTGCCA
	Zo1	AGGACACCAAAGCATGTGAG	GGCATTCCTGCTGGTTACA
	Zo3	ACCCTATGGCCTGGGCTTC	CCCGGGTACAACGTGTCC
	Muc1	GGTTGCTTTGGCTATCGTCTATTT	AAAGATGTCCAGCTGCCCATA
	Muc5ac	Purchased from Qiagen (Qt01196006)	
	Muc6	TGCTCCCAGAATGAGTACTTCGA	CAGAGGTGGAACTGTGAAACTCAGT
	Muc13	GCCAGTCCTCCCACCACGGTA	CTGGGACCTGTGCTTCCACCG
Brain	*Gadph*	TGAAGCAGGCATCTGAGGG	CGAAGGTGGAAGAGTGGGAG
	Rpl	CCTGCTGCTCTCAAGGTT	TGGTTGTCACTGCCTCGTACTT
	Ubc	AGGTCAAACAGGAAGACAGACGTA	TCACACCCAAGAACAAGCACA
	Hprt	AGTGTTGGATACAGGCCAGAC	CGTGATTCAAATCCCTGAAGT
	Cat	ACATGGTCTGGGACTTCTGG	CAAGTTTTTGATGCCCTGGT
	Sod1	AACCAGTTGTGTTGTCAGGAC	CCACCATGTTTCTTAGAGTGAGG
	Sod2	GCCCCCTGAGTTGTTGAATA	AGACAGGCAAGGCTCTACCA
	Gpx1	GAGGGTAGAGGCCGGATAAG	AGAAGGCATACACGGTGGAC
	Gpx2	GAGAACGGCACCAACGAG	TCAGGTAGGCGAAGACGG
	Nrf2	TCTTGGAGTAAGTCGAGAAGTGT	GTTGAAACTGAGCGAAAAAGGC
	Gclm	AGGAGCTTCGGGACTGTATCC	GGGACATGGTGCATTCCAAAA
	Gclc	GGGGTGACGAGGTGGAGTA	GTTGGGGTTTGTCCTCTCCC
	Gsr	CACGGCTATGCAACATTCGC	GTGTGGAGCGGTAAACTTTTTC
	Hmox1	AAGCCGAGAATGCTGAGTTCA	GCCGTGTAGATATGGTACAAGGA
	Nox1	AGAGCCACTGACATCCTGACAG	ACTTGGGGTGGGCAGTAGCTA
	Nox2	CATTCACACTGACCTCTGCTCC	ACAGCCACAAGCATTGAATAGC
	Txn2	TGGGCTTCCCTCACCTCTAAG	CCTGGACGTTAAAGGTCGTCA
	Txndr1	CCCACTTGCCCCAACTGTT	GGGAGTGTCTTGGAGGGAC
